# Quadruple pharmacotherapy for alcohol use disorder tolerable yet insufficient: a case report

**DOI:** 10.1186/s13011-024-00599-6

**Published:** 2024-02-29

**Authors:** Dale Terasaki, Aimee Ellinwood, Dan White

**Affiliations:** 1 Denver Health & Hospital Authority, 777 Bannock St, 80204 Denver, CO USA; 2 Versatile Therapy Solutions, LLC, 80120 Littleton, CO USA

**Keywords:** Alcohol-related disorders, Alcohol pharmacotherapy, Hospital utilization, Involuntary treatment

## Abstract

**Background:**

Combinations of alcohol use disorder (AUD) medications have been investigated, but few if any reports describe patients maintained on more than two options at the same time.

**Case presentation:**

We report a case of a middle-aged man hospitalized with gastrointestinal bleeding and acute kidney injury who had been maintained on four AUD medications (naltrexone, acamprosate, disulfiram, and gabapentin) and multiple psychiatric medications simultaneously as an outpatient. Direct quotations of his experiences with each AUD medication are included, revealing some deviations from what was prescribed as well as nuanced perceptions of effects. Overall, he tolerated the regimen well, but its AUD effects were insufficient to prevent several episodes of returning to alcohol use. He had very high hospital utilization. This prompted the initiation of an involuntary commitment, which began a period of at least six months of sobriety.

**Conclusions:**

Quadruple pharmacotherapy for AUD may be well tolerated and supportive of recovery for an extended period of time. However, for our patient the regimen ultimately failed to prevent multiple episodes of returning to alcohol use and serious medical complications. In refractory cases like this, more intensive interventions such as involuntary commitment can be considered.

## Background

Some 30 million Americans age 12 years and older suffer from alcohol use disorder (AUD), [1] a chronic brain disorder resulting in several functional and physiological consequences [2]. Data suggest that the COVID-19 pandemic increased alcohol-related morbidity and mortality [3, 4] substantially. In the United States, there are three officially approved medications (naltrexone, acamprosate, and disulfiram) for the treatment of AUD. There are also several pharmacotherapies used off-label including gabapentin, a medication shown in some trials to reduce heavy drinking among those who experience alcohol withdrawal symptoms [5]. 

Data exist on combining two medications for AUD, including naltrexone plus acamprosate [6] and naltrexone plus gabapentin [7]. However, few if any published reports exist that describe patients maintained on more than two AUD pharmacotherapy options simultaneously.

We present a case of a middle-aged man who was maintained on four medications for AUD (naltrexone, acamprosate, disulfiram, and gabapentin) and presented to our safety-net hospital for admission. Aspects of his clinical course, alcohol history, pharmacotherapy experience, and subsequent course will be discussed. Written consent from the patient was obtained; some demographic details have been altered to minimize risk of identification.

## Case presentation

### Index hospital admission

Mr. R is a 47-year-old man who presented to the emergency department (ED) with coffee-ground emesis in the context of heavy drinking. He had a self-reported psychiatric history of schizoaffective disorder, attention deficit/hyperactivity disorder, and depression. He had used cocaine and marijuana infrequently in the past.

On initial evaluation, Mr. R’s heart rate was 120 beats per minute, oxygen saturation was 88% on room air, his hemoglobin level dropped from 11.0 to 9.5 g/dL, and his serum creatinine was 1.44 mg/dL (baseline 0.7–1.1), later peaking at 3.01 (glomerular filtration rate, GFR, 25.21 ml/min). He had a liver ultrasound from 3 months earlier that read, “mild hepatomegaly with steatosis. No ultrasound evidence of cirrhosis or portal hypertension.” After acute stabilization with fluid resuscitation, symptom-triggered alcohol withdrawal management, and close monitoring of blood counts, Mr. R underwent an endoscopy, revealing esophagitis and gastritis.

During the admission, he was evaluated by the addiction consult service, [8] who confirmed that his home AUD medications included naltrexone, acamprosate, disulfiram, and gabapentin. They recommended a reduced dose of acamprosate and gabapentin due to a reduced GFR. Disulfiram was not continued as the patient’s adherence appeared to fluctuate, placing him at risk of severe vomiting and bleeding in a future episode of drinking. He had received intramuscular naltrexone two weeks prior, so there was no indication to administer it imminently. Notably, he mentioned taking large amounts of ibuprofen in recent weeks following an outpatient septoplasty procedure.

### Alcohol use disorder history

Mr. R began heavy alcohol use in college, increasing after graduation and finding work as a teacher. At the most, he drank 1.75 L of whiskey per day. This led to numerous problems including losing relationships and jobs (teaching). He had multiple prior hospital encounters related to alcohol withdrawal and other complications. Mr. R sought treatment at the recommendation of his primary care physician (PCP), attending addiction clinic for therapy and medications (co-managed with PCP).

In therapy, Mr. R presented as alert, oriented, and lucid. He described his depression as a “black hole” within his body, though with no active suicidality. Mr. R was highly creative and had been a prolific artist. He lamented about loneliness and boredom. He struggled to process the loss of relationships. Addressing sleep issues, existential issues, triggers leading to use, relapse prevention, and building a sober support network were goals in therapy. He regularly attended appointments, took AUD medications consistently, and engaged in support groups.

**Fig. 1:**
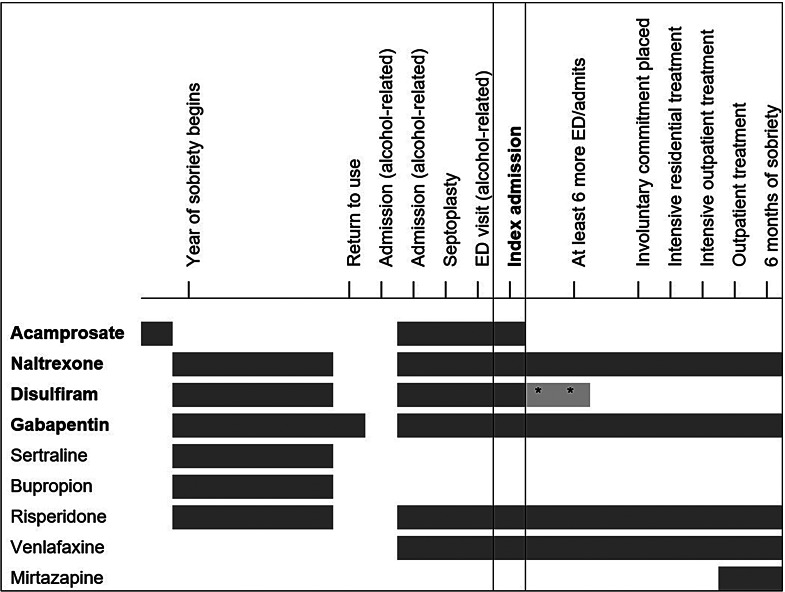
Timeline of recent events as well as alcohol use disorder medications and psychiatric medications. * = disulfiram was not recommended but the patient continued to take on occasion. ED = emergency department. Intervals intended to display sequence, not precise lengths of time

See Fig. 1 for a timeline of more recent events along with prescribed AUD and psychiatric medications. He had a period of sobriety for 12 months, ending a couple months prior to the index admission. For much of this time-period, his AUD medications were intramuscular (IM) naltrexone 380 mg monthly, disulfiram 250 mg daily, and gabapentin 600 mg TID; his psychiatric medications were sertraline 150 mg daily, bupropion 300 mg daily, and risperidone 0.5 mg daily. He had been prescribed acamprosate 666 mg three times daily (TID) prior to his year of sobriety.

Near the end of this sober period, Mr. R’s outpatient therapist had been on leave, he missed a naltrexone injection, he stopped taking his psychiatric medications, and he started a new teaching job– where students were reportedly disrespectful. He identified job frustrations as well as boredom and loneliness as his main triggers to drink multiple 750 ml liquor bottles over 3 days, resulting in a hospitalization for hematemesis (prior to index).

He then started venlafaxine 225 mg daily, increased his risperidone to 2 mg nightly, and restarted acamprosate plus his other AUD medications. He returned to drinking within two weeks, leading to another hospital admission. One month later, he had his septoplasty performed. Two weeks later, he began drinking again and presented to the emergency department, discharging upon rapid detoxification. Another two weeks later, he presented to the hospital for the index admission.

### Experiences with medications

Quotations were recorded with permission during the index admission and displayed in Table 1. Unless otherwise specified, the patient was prompted to describe his experience with a medication and mention any beneficial or adverse effects. Quotations are lightly edited for clarity.

**Table 1:** Mr. R’s reported experiences with medications for alcohol use disorder

Medication	First pre-scribed	Patient’s usual dose	Reported experience
Naltrexone (oral)	2016	50 mg daily	“I think that worked fairly well. I still take it every now and then even when I’ve had the [intramuscular naltrexone]. I think it works for when I start to crave.”
Naltrexone (intra-muscular)	2019	380 mg monthly	“I wouldn’t really feel much…of it, like slowing me down…I don’t know what it was supposed to do. Whatever it’s supposed to do, I don’t think it was doing it and I think I was just doing it. Maybe it works on other people differently, but it definitely didn’t work on me because I just drank a lot if I relapsed like I did this last time, and I don’t know if that’s like a common thing: if you relapse on [intramuscular naltrexone] you drink a lot.”
Acamprosate	2016	666 mg three times daily	“And that really was helpful…my doctor said it was for like cravings and stuff like that.” *Question: Was it very difficult to take it so many times in the day or not really?* “Yeah it was difficult because I would forget to take it like three times a day. It goes two pills and [I’d forget], but I’d eventually like catch up to it or whatever.”
Disulfiram	2021	250 mg daily	“I would use [it] off and on though, because I just felt like I didn’t need to take it because I…wasn’t gonna drink or anything. And then when I had my big relapse in September, I wasn’t taking it either. Well, I was taking a little bit before that, but then I stopped taking it because I was setting myself up to drink and then…when I decided to go ahead and drink, the other self took over. I went ahead and just started drinking and I didn’t get sick or anything because it was like, over 24 h from taking it so, you know, it just wasn’t in my system anymore.” *Question: Have you ever had a disulfiram reaction?* “I don’t think so. I don’t know, I may have because a couple times when I started drinking, I threw up right away. But then I’ll end up continuing to drink.” *Post-encounter correspondence*: “However just to let you know I still am taking [disulfiram]. It is my belief that it helps me deter myself from drinking.”
Gabapentin	2018	600 mg three times daily	“That one helps with like…relaxation and slowing me down. And it helps me to…kind of regain like a little bit of more self-awareness. It did help me focus actually, and to help me feel better. [But] if when I start [taking] it with alcohol though, it just made me tired.”

### Subsequent course

Mr. R followed up with a new counselor. He continued his AUD and psychiatric medications and followed up regularly with his outpatient providers. He presented to the hospital again six times before being placed on a state-sanctioned involuntary commitment (compulsory treatment, typically starting in a residential setting). He was discharged from residential treatment after four weeks to an intensive outpatient program. He continued to engage in outpatient counseling. At six months following initiation of the involuntary commitment, he had not resumed alcohol use.

## Discussion

We reported a case of a man prescribed four simultaneous pharmacotherapies for alcohol use disorder. Overall, this combination of AUD medications was well tolerated and at times appeared to be effective at promoting recovery (i.e., during his sober year). However, the regimen later appeared to be insufficient at preventing Mr. R from resuming alcohol use, highlighting the potentially refractory nature of severe AUD and the importance of concurrent coping skills, structured therapeutic environments, community supports, and treatment of co-occurring psychiatric and medical disorders. Each medication’s moderate effect on AUD did not appear to be additive for Mr. R.

Our patient took certain medications differently than prescribed (Table 1). For example, he took disulfiram and oral naltrexone at times in an *as-needed* manner, [9] and he took gabapentin primarily at bedtime for relaxation/sleep. This underscores that medications– particularly those for substance use disorders– are often self-titrated or self-scheduled to effect. That he considered oral naltrexone to be favorable to intramuscular naltrexone and adamantly wanted to continue disulfiram despite an apparent lack of effectiveness suggests that he derived some psychological or habitual value from daily medication administration. His eagerness to continue these medications despite his recurrent episodes of returning to drinking also emphasizes his desperation to find an effective treatment.

Several combinations of AUD pharmacotherapies have been studied, many of which involve a medication added on to naltrexone therapy. In the landmark, multi-site (*n* = 1383), 8-arm COMBINE trial, [10] the acamprosate plus naltrexone arm (*n* = 157) did not appear to perform better than placebo or naltrexone-alone arms. This is consistent with a 2003 trial (*n* = 160) that observed a comparatively lower relapse rate of this combination compared to placebo or acamprosate alone, but not compared to the naltrexone alone arm [6]. Petrakis et al. tested disulfiram and naltrexone among those with alcohol use disorder and comorbid psychiatric disorders, finding a modest benefit with each agent but no advantage with the combination [11]. Gabapentin was combined with naltrexone in a 2011 trial that secondarily subdivided participants by the presence of alcohol withdrawal symptoms, finding that the combination was particularly effective among those reporting alcohol withdrawal symptoms [7]. Memantine, an N-methyl-D-aspartate receptor antagonist, combined with naltrexone, has been evaluated in a human laboratory, crossover trial, finding that sequentially adding on memantine after initiation of naltrexone led to further reduction of drinking, a finding not observed when the sequence was reversed [12]. A small, non-controlled case series (*n* = 5) of individuals receiving both intramuscular naltrexone and intravenous ketamine showed a reduction in both depressive symptoms and cravings for alcohol [13]. 

This patient was ultimately placed on an involuntary commitment (IC). As of this writing, there are nearly 40 American states that have laws permitting an IC of individuals due to alcohol and/or substance use. Involuntary commitment pertaining to addiction treatment is a serious and controversial topic. Some advocate that, due to a lack of efficacy data and potential for traumatic harm, ICs are often not ethically justified [14]. This is counterbalanced by anecdotal cases– such as with Mr. R– in which pursuing an IC appeared to be the only option to reduce the risk of hospitalization and death related to imminent return to drinking in the context of a tenuous medical status (frequent gastrointestinal bleeding).

In conclusion, this may be the first published report of quadruple therapy for AUD (naltrexone, acamprosate, disulfiram, and gabapentin). It may be well tolerated and effective at times for maintaining AUD recovery. Unfortunately, the anti-craving or protective effects of combination pharmacotherapy can certainly be overcome by other psychosocial factors, leading to resumption of alcohol use and serious medical complications. In these cases, other approaches– including involuntary commitment– can be considered.

## Data Availability

Not applicable.

## Abbreviations

AUD: alcohol use disorder
ED: emergency department
PCP: primary care provider/physician
TID: three times daily
IC: involuntary commitment
